# New Genetic Technologies in Alcohol Research

**Published:** 1997

**Authors:** Gregg E. Homanics, Susanne Hiller-Sturmhöfel

**Affiliations:** Gregg E. Homanics, Ph.D., is an assistant professor in the Departments of Anesthesiology/Critical Care Medicine and Pharmacology, University of Pittsburgh, Pittsburgh, Pennsylvania. Susanne Hiller-Sturmhöfel, Ph.D., is a science editor of Alcohol Health & Research World

**Keywords:** molecular genetics, hereditary factors, gene, AOD dependence, research and evaluation, method, animal strains, animal model, serotonin receptors, GABA (gamma-aminobutyric acid) receptors, protein kinases, NMDA (*N*-methyl-d-aspartate) receptors, AODE (alcohol and other drug effects), liver, immune system, laboratory study, literature review

## Abstract

Recently developed approaches to creating genetically engineered animals have expanded researchers’ repertoire of methods to investigate the roles of individual genes in the development of certain behaviors and diseases, including alcoholism. For example, knockout mice, in which single mouse genes have been inactivated, have allowed scientists to assess the roles of those genes in mediating some of alcohol’s effects. This approach has been further refined using conditional gene knockout technology, which allows the inactivation of a gene only in certain cells or during specific developmental periods. Alternatively, transgenic mice (i.e., mice that carry a foreign gene in addition to their own genes) have been created in which researchers can activate or inactivate the foreign gene at will. Although these genetic engineering technologies have not yet been used extensively in alcohol research, they offer great promise in analyzing the functions of genes that may be involved in determining alcohol’s effects on the body and the development of alcoholism.

Alcohol researchers frequently use animal models instead of humans to study alcohol-drinking behavior, alcohol’s effects on the body, and the mechanisms underlying the development of alcoholism. Animal models offer several advantages over analyses in humans or cultured cells for studying the effects of alcohol. For example, selective breeding techniques have generated numerous strains of mice and rats that exhibit particularly high or low levels of alcohol consumption or altered sensitivity to certain alcohol effects. By studying these animals, researchers can assess responses to alcohol on a molecular, cellular, and even behavioral level and compare the results of such analyses among different rodent strains.

Based on these analyses as well as on studies in human alcoholics, researchers have identified various genes (i.e., candidate genes) that may play a role in mediating alcohol’s effects on the brain and other organs or that may contribute to the development of alcoholism. Some of these candidate genes contain the information for (i.e., encode) neurotransmitter receptors^1^—proteins located on nerve cells, or neurons, that interact with brain chemicals involved in the communication between neurons (i.e., neurotransmitters). Other candidate genes encode proteins involved in the transmission of signals within the cells. The next challenge for scientists is to investigate the contribution of each of these candidate genes in modifying the body’s responses to alcohol. The ultimate objective of this research is to understand how these genes influence behavior. Animal models can help scientists achieve that goal.

Recent advances in the techniques for creating genetically engineered animals—mostly mice—offer alcohol researchers exciting opportunities to analyze the physiological roles of candidate genes in the body’s responses to alcohol. These new techniques include the generation of both “knockout mice” and transgenic mice. In knockout mice, one of the animal’s genes is inactivated. In transgenic mice, in contrast, an active foreign (e.g., human) gene is permanently integrated into the animal’s DNA. Both of these approaches allow researchers to analyze the effects of individual genes on behavior or disease development. This article describes some of the methods used to generate these genetically engineered mice and the limitations of these approaches. In addition, the article reviews a few examples demonstrating the potential of such animal models for elucidating some of the mechanisms mediating the effects of alcohol and underlying the development of alcoholism.

## Conventional Gene Knockout Technology

A powerful approach to analyzing the function of a specific gene and determining its role in the development of a certain disorder (e.g., alcoholism) is to inactivate (i.e., knock out) the gene in the intact organism and study the consequences of this modification on disease development or behavior. By comparing the appearance or behavior (i.e., the phenotype) of knockout mice to that of normal (i.e., wild-type) mice, researchers can draw inferences about the function of the gene under investigation (i.e., gene X).

### Generation of Knockout Mice

**Figure 1:**
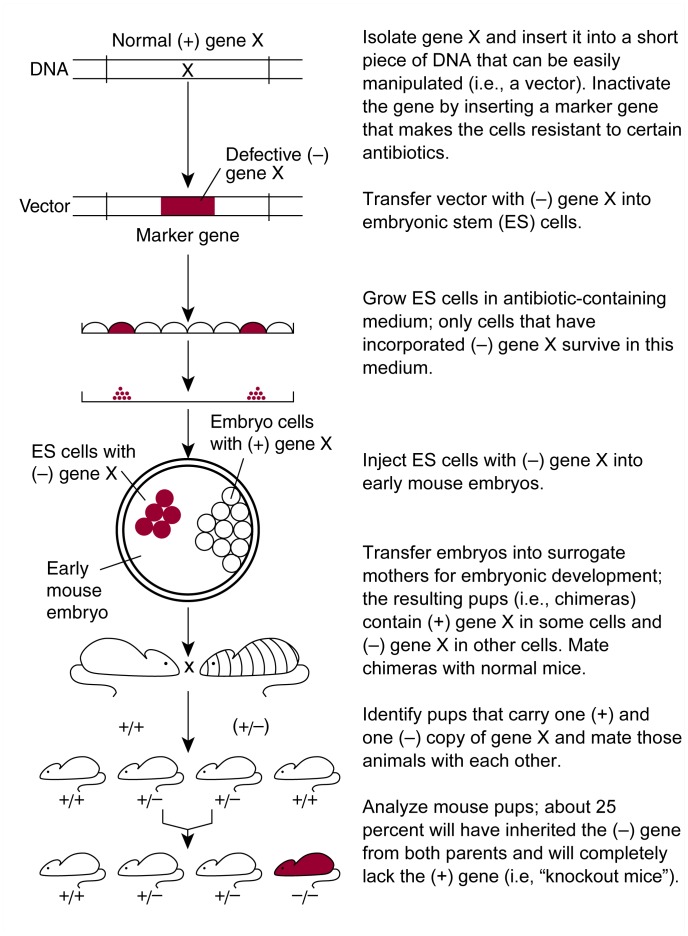
Strategy for generating conventional knockout mice.

The process of creating knockout mice requires multiple steps to obtain genetically engineered animals in which both copies of gene X (i.e., the copy inherited from the mother and the copy inherited from the father) are nonfunctional (Capecchi 1994; Galli-Taliadoros et al. 1995). To inactivate gene X, researchers must first isolate the gene from mouse DNA, transfer it into a short piece of DNA that can be manipulated easily (i.e., a vector), and determine the gene’s exact structure. Next, a change (i.e., a mutation) is introduced into the gene’s structure. Most commonly, scientists insert another gene—a so-called marker—into gene X (see figure 1). Marker genes often encode proteins that make a cell resistant to certain antibiotics. This modification serves two purposes. First, it disrupts gene X so that the gene no longer produces a functional protein (i.e., gene product). Second, when the vector containing the mutated gene X is introduced into cells, the marker allows the cells to survive when grown in a solution (i.e., medium) containing an antibiotic. In contrast, cells that do not take up the marker-containing gene X die when exposed to the antibiotic.

In a tissue-culture dish (i.e., in vitro), the vector with the mutated gene X then is transferred into a specific type of mouse cell called an embryonic stem (ES) cell. These cells, which in the intact animal can develop into all of the different cell types and tissues that make up a mouse, also contain gene X. Not all ES cells actually take up the vector. Those that do can be identified by growing the cells in the antibiotic-containing medium, because only cells that contain the marker can survive and multiply in this medium. In a small percentage of the ES cells that take up the vector, the normal gene X exchanges places with the mutated gene X through a process called homologous recombination. Using molecular biological techniques, scientists identify the few ES cells that have undergone homologous recombination among the others that have taken up the vector.

Next, the modified ES cells are injected into mouse embryos at an early stage of development. Because embryos cannot develop to term in vitro, they are then implanted into surrogate mothers. The injected ES cells and the embryo’s own cells together develop into an intact mouse. These animals are called chimeras, because some of their cells are derived from the ES cells and contain the mutated gene X, whereas other cells are derived from the embryo’s cells and contain the normal gene X. By selecting specific mouse strains for these experiments, researchers can easily identify the chimeras: If, for example, the ES cells are derived from a mouse strain with a black coat and the embryos are derived from a mouse strain with a white coat, chimeric mice will be characterized by black and white spots or stripes. Conversely, mice that do not contain the mutated gene X (e.g., because the injected ES cells did not survive in the embryo) will be all white.

The chimeric mice do not contain the mutated gene X in all of their cells. Only some of the animals carry the modified gene in their reproductive (i.e., germ) cells and therefore can pass it on to their offspring. To identify these animals, the chimeric mice are mated with wild-type mice containing only the functional gene X. Any offspring carrying the mutated gene X will have inherited it from the chimeric parent. In these offspring, the mutated gene X is present in all the cells in their body. In addition, these animals carry a normal copy of gene X that they have inherited from their wild-type parent. To obtain animals in which both the maternal and the paternal copies of gene X are mutated, two of these animals are mated with each other. According to the basic laws of inheritance, approximately 25 percent of the offspring will carry two copies of the mutated gene X. These animals, which can be identified using molecular biological techniques, are the knockout mice whose responses or behavior can then be analyzed.

### Examples of Conventional Knockout Technology

With more and more candidate genes being identified, researchers also have begun to use knockout mice to analyze how these genes contribute to the development and manifestations of alcoholism. Most of the relevant knockout mice generated to date carry inactivated neurotransmitter receptors (e.g., the receptors for the neurotransmitters serotonin and gamma-aminobutyric acid [GABA]) or mutated versions of molecules involved in the transmission of signals within cells (e.g., protein kinase C [PKC] and a protein kinase called fyn).

#### Serotonin-Receptor Knockout Mice

Serotonin is a neurotransmitter that subtly modifies the function of neurons. It exerts its effects by interacting with receptors on the neuron’s surface. Serotonin-mediated neurotransmission may play a role in mediating alcohol’s effects on the brain and may contribute to the development of alcohol abuse. For example, researchers have found that alcoholics appear to have lower serotonin levels in their brains than do nonalcoholics (Virkunnen et al. 1995). Moreover, alcohol exposure affects the function of serotonin receptors, and medications that act on these receptors alter alcohol consumption in humans and animals (Pettinati 1996).

To investigate the relationship between alcohol consumption and serotonin-receptor function in more detail, researchers have analyzed knockout mice that lack one specific serotonin-receptor subtype (i.e., the 5-HT_1B_ receptor) with respect to the animals’ alcohol consumption level and responses to alcohol (Crabbe et al. 1996). These analyses found that the 5-HT_1B_ knockout mice voluntarily drank about twice as much alcohol as did their wild-type counterparts. In addition, the knockout animals were less likely than wild-type mice to lose motor coordination after drinking alcohol. They also developed tolerance to alcohol’s incoordinating effects much more slowly than did wild-type mice. Knockout and wild-type mice did not differ, however, in the severity of their withdrawal symptoms when alcohol was withheld. These studies demonstrate that the 5-HT_1B_ receptor plays a role in regulating alcohol consumption and contributes to some of alcohol’s effects. Moreover, the findings suggest that tolerance and withdrawal do not involve the same receptors.

#### GABA_A_-Receptor Subunit Knockout Mice

Some neurotransmitters (i.e., inhibitory neurotransmitters) reduce the excitability of neurons, whereas others (i.e., excitatory neurotransmitters) increase the excitability of neurons. High activity of inhibitory neurotransmitters can lead to incoordination, sedation, and anesthesia. GABA is the major inhibitory neurotransmitter in the central nervous system (CNS); its actions are mediated primarily by the GABA_A_ receptor. Each functional GABA_A_ receptor consists of five protein molecules (i.e., subunits). The different subunits fall into five categories: α, β, γ, δ, and ɛ subunits.

Because alcohol consumption causes motor incoordination and sedation, researchers have suspected that GABA and the GABA_A_ receptor contribute to alcohol’s effects on the brain. Moreover, several studies have indicated that alcohol’s effects on the GABA_A_ receptor play an important role in the development of alcohol tolerance and alcohol dependence. The GABA system also may be involved in determining a person’s susceptibility to developing alcohol abuse and dependence (Mihic and Harris 1997). To investigate these potential associations in more detail, scientists have created knockout mice specifically lacking individual GABA_A_ receptor subunits (i.e., the α6 and the β3 subunits). These two subunits were chosen because various studies using cultured cells, tissue samples (i.e., in vitro studies), and animal models (i.e., in vivo studies) have suggested that alcohol, sedating medications, and anesthetics affect GABA_A_ receptors containing these subunits.

The α6 knockout mice were compared with wild-type mice with respect to several responses to alcohol. These responses included the extent of alcohol’s hypnotic effects (i.e., how long the mice slept, or passed out, after receiving an alcohol injection), tolerance to alcohol’s incoordinating effects, severity of withdrawal seizures, and degree to which the mice developed tolerance to alcohol’s sedating effects if they had previously received alcohol (Homanics et al. 1997, 1998). Surprisingly, the knockout mice did not differ from the wild-type mice with respect to any of these variables. Similarly, β3 knockout mice exhibited no change in sleep time in response to alcohol (Quinlan et al. 1998). Thus, in contrast to previous studies, these in vivo studies in intact genetically engineered animals appear to indicate that the α6 and β3 subunits do not play a key role in these specific responses to alcohol. The studies do not conclusively establish that these genes are completely irrelevant, however, because other responses, such as the level of alcohol consumption, have not yet been tested in these mice. Alternatively, because the knockout mice lacked the inactivated α6 or β3 subunits throughout their development, other subunits may have compensated for their absence, thereby obscuring any effects resulting from the inactivation of the subunits. This and other limitations of the conventional knockout approach are discussed in more detail later in this article.

#### PKC Knockout Mice

PKC is one of a group of enzymes called protein kinases, which add phosphate groups to other proteins in a chemical reaction called phosphorylation. Phosphorylation can activate or inactivate the modified proteins. Protein phosphorylation by PKC plays an important role during the transmission of signals within cells. One protein whose function may be regulated by PKC is the GABA_A_ receptor. Several in vitro studies have indicated that phosphorylation by PKC enhances the GABA_A_ receptor’s sensitivity to alcohol and may also affect the receptor’s activity in response to other sedatives, such as barbiturates.

To investigate the relevance of these processes in vivo, Harris and colleagues (1995) used knockout mice that lacked a PKC variant called PKCγ. PKCγ is present only in the CNS, including certain cells that appear to be important for some of alcohol’s behavioral effects (e.g., alcohol’s hypnotic effect). These analyses found that the knockout mice were less sensitive to alcohol’s hypnotic effect, because the animals did not sleep as long after receiving an alcohol injection as did wild-type mice. The reactions of the knockout mice to several other sedatives that affect the GABA_A_ receptor, however, were not altered. Similarly, extracts of brain cells from the knockout mice exhibited reduced enhancement of GABA_A_ function in response to alcohol but not in response to the other sedatives. These findings suggest that PKCγ plays a pivotal role in mediating alcohol’s effects on the GABA_A_ receptor in vivo as well as in vitro. In addition, the results demonstrate that other sedatives affect the GABA_A_ receptor through a different mechanism from that of alcohol.

#### Fyn Kinase Knockout Mice

A protein kinase called fyn kinase also phosphorylates neurotransmitter receptors in the brain. One of these receptors is the *N-*methyl-d-aspartate (NMDA) receptor, which interacts with the neurotransmitter glutamate. Alcohol also modulates the function of the NMDA receptor, and this modulation may be one of the pathways through which alcohol exerts some of its behavioral effects. Using knockout mice lacking the fyn gene, Miyakawa and colleagues (1997) investigated whether this kinase is involved in determining sensitivity to alcohol’s hypnotic effects. The researchers administered alcohol to fyn knockout mice and control wild-type mice and then performed a sleep-time assay: They placed the intoxicated animals on their backs and measured the time it took them to stand up again. These analyses found that the knockout mice generally took twice as long to get back on their feet after alcohol administration as did the wild-type mice. Because the knockout and wild-type mice did not differ in their responses to another sedative, the researchers determined that the effect was specific to alcohol.

The recovery from alcohol’s hypnotic effects appears to be associated with enhanced phosphorylation of the NMDA receptor because 5 minutes after alcohol administration, when the wild-type mice began to recover, their levels of NMDA-receptor phosphorylation in a brain region called the hippocampus increased. This region, which plays a role in learning and memory, also may be involved in alcohol’s hypnotic effects. Conversely, the knockout mice, which recovered more slowly, exhibited no increase in NMDA-receptor phosphorylation in the hippocampus. These findings suggest that fyn kinase and the NMDA receptor play crucial roles in the animals’ sensitivity to alcohol.

### Limitations of Conventional Knockout Technology

The results of the studies previously described demonstrate that the targeted inactivation of candidate genes can allow researchers to analyze the roles of these genes in mediating alcohol’s effects on the brain. As indicated by the example of the GABA_A_-receptor knockout mice, however, the results of these in vivo studies are not always predictable or consistent with the findings from in vitro analyses. Although these inconsistencies may indicate that in vitro analyses do not always adequately represent reactions in an intact organism, they also may result from experimental limitations of the knockout technology. These limitations include the following:

- Many genes are essential for the development of the organism. Consequently, mouse embryos are not viable when such genes are inactivated.
- Many behaviors and disorders (e.g., alcoholism) are determined by the activities and interactions of numerous genes, rather than a single gene. Accordingly, the effect of disrupting just one gene may not be detectable or may involve only one aspect of the disorder under investigation. In addition, the activities of other genes may change in response to the inactivation of one gene, thereby masking any effect the gene inactivation may have had. In either scenario, researchers would observe no change in the knockout mice compared with wild-type mice, thereby failing to identify the function of the inactivated gene.
- Many genes are active in numerous cells in the body but affect a certain behavior only through their activity in some cells (e.g., specific brain regions). Because the genes in knockout mice are inactivated in all cells, it is impossible to determine the exact location of the cells that are actually involved in controlling the phenotype studied.

To overcome some of these limitations, researchers have developed new approaches to generating knockout mice. In these approaches, the genes are inactivated only in specific cells or during certain stages in the animal’s life. The most important of these new strategies is discussed in the following section.

## Conditional Gene Knockout Technology

To limit the inactivation of candidate genes to certain cell types or developmental stages, researchers use a two-component “gene-cutting” system, called the Cre-loxP recombination system (Rajewsky et al. 1996). The components of this system are derived from a virus that only infects bacteria. This virus produces an enzyme—Cre—that cuts out (i.e., excises) from the cell’s DNA any sequences that are located between two copies of a short DNA motif called a loxP site. Both the gene encoding the Cre enzyme and the loxP sites have been isolated and can be introduced into vectors for easy manipulation.

**Figure 2:**
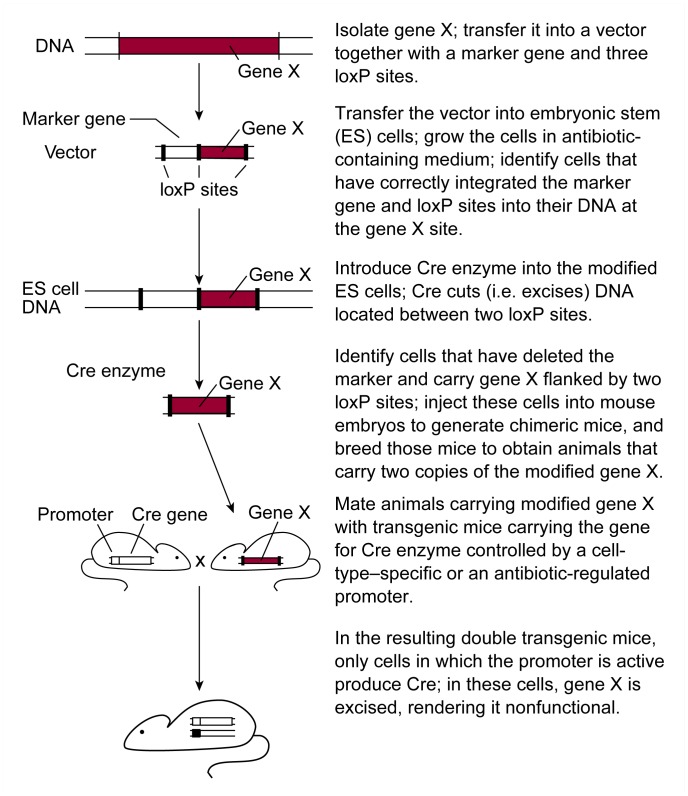
Strategy for generating conditional knockout mice. SOURCE: Adapted from Galli-Taliadoros et al. 1995.

To use the Cre-loxP system for generating knockout mice, two different strains of genetically engineered mice must be generated (see figure 2). One strain includes transgenic mice that carry the gene encoding Cre in their cells. (The generation of transgenic mice is discussed more fully later in this article.) The activity of the Cre gene is controlled by a molecular “on/off” switch (i.e., promoter). Promoters are DNA sequences that in most cases are located on the chromosome in front of the gene that they control. Numerous promoters exist, because in the intact organism not all genes are active all the time and in all cells. Thus, some promoters are active (i.e., switched “on”) only in specific cells (e.g., neurons) or at specific times during development (e.g., in the adult). Other promoters are active only if the cell or animal is exposed to substances called inducers, such as certain antibiotics (e.g., tetracycline). Cre protein only can be produced if the promoter controlling the Cre gene is switched on. No Cre protein is produced in cells in which the promoter is switched “off” (i.e., inactive), for example, because the animal has not been exposed to the inducer. Numerous strains of transgenic mice can be created, each with a different promoter controlling the Cre gene.

The second mouse strain needed for these analyses carries gene X, flanked by two loxP sites. These mice are generated through mechanisms similar to those involved in standard knockout technology. First, a vector is engineered that contains gene X and a marker gene, with three loxP sites placed in front of, behind, and between those two genes (see figure 2). This vector is introduced into ES cells, and some cells incorporate it into their DNA by homologous recombination. These modified ES cells can be identified as described previously. Because the investigator ultimately wants to obtain cells that contain only gene X flanked by loxP sites, but not the marker gene, Cre protein is introduced into the modified ES cells. As mentioned previously, Cre protein excises DNA segments that are flanked by two loxP sites. The modified ES cells contain three loxP sites. The excision process therefore can result in three different products: (1) cells in which only the marker gene has been deleted, (2) cells in which only gene X has been deleted, and (3) cells in which both the marker gene and gene X have been deleted. The investigator then identifies those ES cells that have lost only the marker gene and which now contain gene X flanked by two loxP sites. Because loxP sites are small and have been placed in unimportant regions of gene X, the gene continues to function normally. The modified ES cells then are used as described previously to generate chimeric mice and, eventually, mice that carry two functional copies of gene X flanked by loxP sites.

To inactivate gene X in these mice, the animals are mated with the transgenic mice carrying the Cre gene. Some of the resulting offspring carry both the Cre gene and gene X with its adjacent loxP sites. In these animals, cells in which the promoter controlling the Cre gene is active produce Cre protein. Cre protein, in turn, excises gene X, thereby rendering it nonfunctional. In all other cells, however, the promoter controlling the Cre gene is inactive, no Cre protein is produced, and gene X remains intact and functional. For example, if the Cre gene is controlled by a promoter that is activated by an inducer (e.g., an antibiotic), gene X is excised only once the animals are exposed to the antibiotic. Similarly, if the Cre gene is controlled by a promoter that is active only in liver cells, gene X will be inactivated only in those cells.

By generating numerous strains of transgenic mice, each of which carries the Cre gene controlled by a different promoter, researchers theoretically could use the knockout approach to study the functions of genes in a wide variety of cells or tissues or during various developmental stages. To date, few Cre-transgenic mouse strains exist, but their numbers are rapidly increasing as many researchers are making use of this powerful technological advance.

### Examples of Conditional Knockout Mice

Conditional gene targeting has not yet been used in alcohol research; however, existing examples of this technology may allow future alcohol-related applications. For example, researchers investigating alcohol’s effects on the brain want to analyze the functions of genes in specific brain regions that may be involved in the many effects of alcohol. Such studies would greatly benefit from methods in which specific genes are inactivated only in certain brain regions.

Conditional gene targeting using a Cre gene controlled by a selectively acting promoter will allow researchers to analyze the functions of specific genes in distinct areas of the brain. The feasibility of this approach was recently demonstrated by Tsien and colleagues (1996*a*,*b*). These investigators generated transgenic mice carrying a Cre gene with a promoter that is inactive until after birth and then is active only in some brain regions, including the hippocampus. As mentioned previously, the hippocampus plays an essential role in learning and memory formation and has been shown to be affected by alcohol. The researchers generated several lines of these Cre-transgenic mice that differed slightly in the specific regions of the hippocampus (i.e., the CA1 region, the CA3 region, and the dentate gyrus) in which the Cre gene was activated. These animals then were mated with mice carrying the gene for a certain type of NMDA receptor (i.e., the NMDA1 receptor), which was flanked by loxP sites. In the resulting knockout mice, the NMDA1-receptor gene was excised only in cells of those regions of the hippocampus in which the Cre gene was activated. The knockout mice then were tested for their ability to learn a task that required spatial memory (i.e., remembering the location of a submerged platform in a water tank). These analyses found that animals in which the NMDA1-receptor gene had been deleted specifically in the hippocampal CA1 region exhibited impaired spatial memory compared with wild-type animals. These findings demonstrate that conditional gene targeting approaches can help analyze the functions of genes in brain regions relevant to alcohol research.

## Conventional Transgenic Technology

In contrast to knockout technology, which results in the elimination or inactivation of a gene in the animal, transgenic technology is based on the permanent integration of a foreign gene into an animal’s DNA (Wehner and Bowers 1995). This technique allows researchers to evaluate the role of that gene and its protein product during fetal development or to model human diseases in animals. For example, researchers have transferred into mice those genes that are involved in the development of disorders such as cystic fibrosis in order to study the mechanisms underlying the disease in more detail.

**Figure 3:**
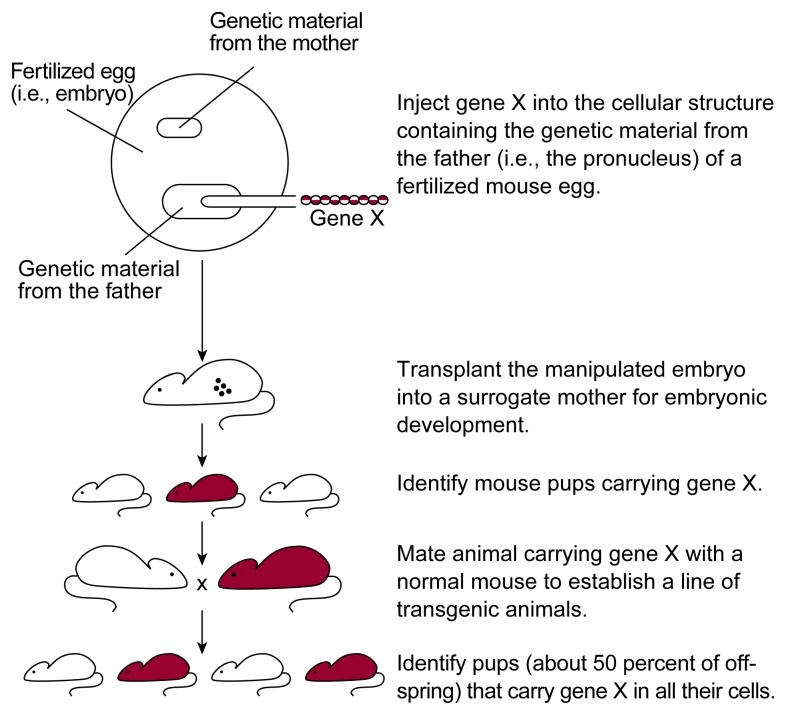
Strategy for generating transgenic mice.

To generate mice that are transgenic for gene X, the gene must be isolated from its natural source (e.g., human DNA) and inserted into a vector. The vector then is injected with a very fine needle into a fertilized mouse egg (see figure 3). These single-cell eggs, or embryos, contain two structures (i.e., pronuclei), each containing one set of DNA—one inherited from the mother (i.e., the female pronucleus) and one inherited from the father (i.e., the male pronucleus). The vector carrying gene X is typically injected into the larger male pronucleus. In a small percentage of embryos, the foreign gene randomly integrates into one of the embryo’s chromosomes. The injected embryos subsequently are transferred into surrogate mothers. Of the resulting mouse pups, those that test positive for gene X are then mated with wild-type mice to establish lines of genetically engineered mice. These transgenic mouse lines can be compared to wild-type mice for the activity of gene X and for the particular phenotype under investigation (e.g., the response to alcohol).

### Examples of Conventional Transgenic Technology

Researchers only recently have begun using transgenic mice to investigate alcohol-related issues, such as alcohol’s effects on the liver and on the immune system. In addition, transgenic mice generated for non-alcohol–related studies have led to findings that are relevant to alcohol research.

#### Analysis of Alcohol’s Effects on the Liver

An important consequence of long-term alcohol consumption in humans is the development of cirrhosis of the liver. One indicator of cirrhosis is the increased production in the liver of the protein called type I collagen. The collagen molecules form fibers of scarred, nonfunctional liver tissue. Collagen production is regulated by the activity of the promoter controlling the collagen gene. In contrast to humans, alcohol administration in rodents by itself does not induce the formation of collagen fibers. This finding suggests that the collagen gene promoter in humans is activated by alcohol, whereas the collagen gene promoter in rodents is not.

To investigate this hypothesis, Walton and colleagues (1996) generated transgenic mice that carry a marker gene controlled by a collagen type I gene promoter isolated from rats. In contrast to most other promoters, the DNA sequences constituting the promoter of the collagen type I gene are located both in front of and within the gene. For their transgenic animals, the researchers only used those promoter sequences that are located in front of the type I collagen gene. The transgenic mice then were treated with alcohol for 4 weeks, and the activities of the collagen promoter-controlled marker gene and of the animals’ own collagen type I gene were assessed. These analyses found that the activity of the marker gene, but not of the animal’s own collagen gene, was markedly increased after alcohol treatment. This finding suggests that even in rodents, the promoter elements located in front of the collagen gene are sensitive to alcohol treatment. Thus, other regulatory elements (e.g., those located within the type I collagen gene) appear to account for the differences between humans and rodents in collagen production in response to alcohol. Moreover, the transgenic animals may allow researchers to investigate alcohol’s effect on the type I collagen gene and on the formation of collagen fibers in more detail in the rodent model.

#### Analysis of Alcohol’s Effects on the Immune System

Numerous studies have indicated that excessive alcohol consumption impairs various functions of the immune system (for a review, see Szabo 1997). One type of immune response affected by alcohol is called delayed hypersensitivity (DH) response. This type of immune response occurs several hours to days after the organism has been exposed to a foreign molecule (i.e., antigen).^2^ The DH response is mediated by a certain type of immune cell called T cell. These cells recognize the specific antigen used, interact with the antigen, and initiate a series of events that result in the antigen’s elimination from the body. To study DH in an animal model, the animal usually must be injected twice with the antigen: once to stimulate the production of T cells and a second time to cause those T cells to initiate the DH response. This two-injection process, however, makes it difficult to assess the DH response. The need for a second antigen injection could be eliminated if the animals already contained sufficient T cells to initiate the DH response after the first antigen injection. Moreover, if the number of T cells that specifically recognize the antigen could be increased, the resulting DH response would be stronger. This enhancement would make it easier to detect alcohol’s effects on the DH response.

Researchers have used transgenic mice to develop an experimental system in which the DH response could be studied in animals after a single antigen injection (Schodde et al. 1996). As an antigen to induce a DH response, the scientists wanted to use a chicken protein called ovalbumin. Accordingly, they generated transgenic mice carrying a gene that enabled most of the animals’ T cells to recognize ovalbumin. When these transgenic mice were injected with ovalbumin, they immediately developed a DH response without requiring a second injection. The researchers then evaluated alcohol’s effects on the DH response to ovalbumin in the transgenic animals. Mice that had received an alcohol-containing diet for 10 days showed a significantly impaired DH response compared with animals that did not receive alcohol. The findings indicate that the transgenic mice represent a valid model to study alcohol’s effect on this type of immune response. For example, researchers can use these animals to investigate in more detail how alcohol interferes with the DH response and how alcohol affects other functions of T cells.

#### Findings From Non-Alcohol–Related Transgenic Mice

In experiments unrelated to alcohol research, Hilakivi-Clarke and colleagues (1992, 1993) studied transgenic mice that produced excessive levels of human transforming growth factor alpha (TGF-α). This protein plays an important role during development; its overproduction, however, can cause liver tumors. Analyses of male transgenic mice carrying the human TGF-α gene found that these animals not only developed liver tumors but also were more aggressive than were wild-type mice. Moreover, the transgenic mice displayed other behaviors indicating an altered function of the neurotransmitter serotonin, which, as previously mentioned, may mediate some of alcohol’s effects. During an experiment to analyze the link between aggression and the development of liver tumors, the researchers also evaluated the animals’ behavioral response to alcohol. These analyses found that compared with wild-type animals, the transgenic mice were more sensitive to some of alcohol’s effects, including its hypnotic effects. Thus, although the TGF-α transgenic mice were created for purposes completely unrelated to alcohol research, they may help scientists learn more about the mechanisms underlying physiological sensitivity to alcohol and about the role of the serotonin neurotransmitter system in these processes.

### Limitations of Conventional Transgenic Technology

Although transgenic mice generated by the conventional technology described earlier can provide important information about the functions of individual genes, this experimental approach also is associated with several limitations, as follows:

- The site in the DNA where gene X integrates cannot be controlled. Consequently, gene X may be surrounded by DNA sequences that regulate gene X activity and allow its activation only in certain tissues or at specific times during development. Accordingly, researchers often must analyze many transgenic mouse lines to identify one with the desired pattern of gene X activity. Moreover, integration of gene X may disrupt a natural gene in the DNA of the transgenic mouse, and the observed phenotype of the animal actually may be the result of this gene’s disruption.
- The amount of the gene X product in the cells cannot be predicted. The observed phenotype, however, may vary, depending on the amounts of the gene product in each cell.
- For genes that are strictly regulated during embryonic development, their expression at the wrong time or in altered amounts may interfere with normal development. The resulting transgenic mice may not be viable, or the observed phenotype may be solely the result of alterations in normal development.

To overcome these limitations, researchers are devising methods to activate the foreign gene in transgenic mice only at specific times during the animal’s life or only in specific cells. One example of these novel approaches is discussed in the following section.

## Regulatable Transgenic Technology

**Figure 4:**
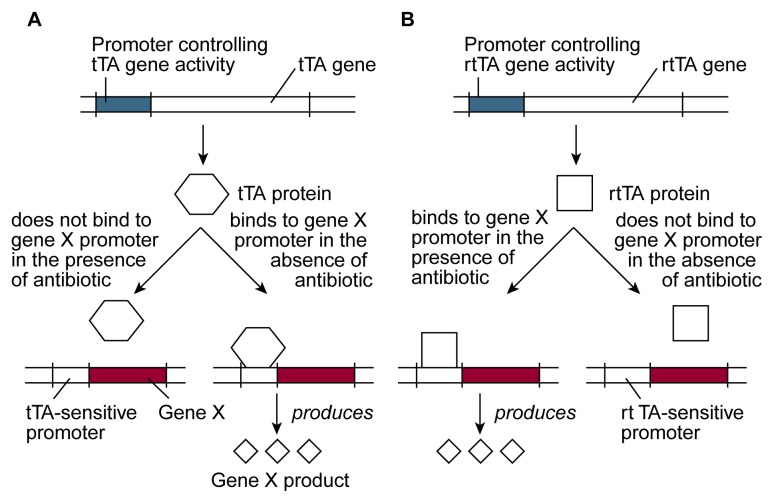
Schematic representation of an antibiotic-sensitive system to regulate the activity of a foreign gene (i.e., gene X) in transgenic mice. The activity of gene X in these animals is governed either by a protein called tetracycline-controlled transactivator (tTA) (A) or by a protein called reverse tetracycline-controlled transactivator (rtTA) (B). The genes encoding tTA or rtTA are introduced into the animals together with gene X. By adding an antibiotic to (or removing it from) the animals’ drinking water, one can control whether tTA and rtTA interact with the promoter that regulates the activity of gene X. Binding of tTA or rtTA to the promoter activates gene X, resulting in the production of the protein encoded by gene X. SOURCE: Adapted from Kistner et al. 1996.

Researchers recently have developed a regulatory system that allows them to control when and in which cells a foreign gene becomes activated (Kistner et al. 1996). This system includes three major components (see figure 4):

- A protein called the tetracycline-controlled transactivator (tTA), which can regulate the function of certain promoters. In the presence of certain antibiotics (i.e., tetracycline and doxycycline), tTA cannot interact with these promoters, and both the promoters and the genes they control are inactive. In the absence of these antibiotics, however, tTA interacts with the promoters. This interaction activates the promoters and, consequently, the genes controlled by them. (An alternative regulatory system uses a protein called reverse tetracycline-controlled transactivator [rtTA], which has the opposite effect: It activates the promoter in the presence of the antibiotics and inactivates the promoter in their absence.)
- The antibiotic—most commonly doxycycline—that regulates whether tTA or rtTA can interact with a promoter.
- Gene X under the control of a promoter that can be regulated by tTA or rtTA.

To use this regulatory system, researchers generate transgenic mice that carry both the gene for tTA (or rtTA) and gene X. Gene X is under the control of a tTA- (or rtTA-) sensitive promoter. The activity of gene X in these transgenic mice can be regulated by adding or removing low, nontoxic doses of the antibiotic (e.g., doxycycline) from the animals’ drinking water. In animals carrying the tTA gene, addition of doxycycline inactivates gene X, and removal of doxycycline activates the gene. In animals carrying the rtTA gene, the antibiotic has the opposite effects. Although long-term exposure to these concentrations of doxycycline does not harm the animals, researchers generally choose the regulatory system that allows them to minimize antibiotic treatment. Thus, they will use rtTA if they want to activate gene X only temporarily and use tTA if they want to inactivate gene X temporarily.

To study the effects of activating or inactivating gene X only in certain cells (e.g., liver cells), one can place the tTA (or rtTA) gene under the control of a promoter that is active only in these cells. As a result, tTA (or rtTA) is produced only in the liver cells and not in other tissues in the body. Because the activity of gene X requires the presence of tTA or rtTA, the product of gene X also can be generated only in the liver cells; in all other cells of the body, gene X will be inactive.

Several factors other than the promoter that controls tTA or rtTA also can affect the activity of gene X. For example, the concentration of the antibiotic in the animal’s drinking water determines the extent to which gene X activity can be increased or decreased. In addition, the locations in the mouse DNA where gene X and the tTA (or rtTA) gene have integrated may influence the extent to which the activity of gene X can be altered. These locations also may determine the specific cells in which the foreign genes are active (e.g., all cells in a brain region, such as the hippocampus, or only a subset of cells in that region).

### Example of Regulatable Transgenic Technology

Alcohol researchers have not yet applied these latest advances in transgenic technology. Studies from other fields, however, suggest that this approach is feasible for studying the genes involved in mediating some of alcohol’s effects (e.g., its effects on thinking and information processing). For example, Mayford and colleagues (1996) have used a tTA-regulated transgenic system to study the role of a gene called calcium-calmodulin-dependent kinase II (CaMKII) in memory formation. The researchers studied the gene’s effects on two types of memory: explicit memory, which is a memory for facts, places, and events, and implicit memory, which is a memory for perceptions (e.g., fear) and motor skills. The formation of explicit memories primarily involves nerve cells in the hippocampus, whereas the formation of implicit memories involves various brain regions, including structures called the amygdala and the striatum.

The researchers generated several transgenic mice carrying (1) a tTA gene under the control of the CaMKII promoter, which is active in various brain regions contributing to memory formation, and (2) the CaMKII gene under the control of a tTA-sensitive promoter. In the absence of doxycycline, these mice should produce increased amounts of the CaMKII gene product in all the cells where it is normally produced. Conversely, in the presence of doxycycline, CaMKII production in these cells should be prevented. When the investigators analyzed the brains of several transgenic mice for the presence of the CaMKII gene product, they found some animals with moderate amounts of the protein in the hippocampus, amygdala, and striatum. Other animals, however, exhibited high levels of CaMKII protein in the amygdala and striatum, but not in the hippocampus.

The animals then were tested for their abilities to form explicit and implicit memories. In the absence of doxycycline, when CaMKII was produced in the cells, animals producing moderate amounts of the protein in the hippocampus failed to form explicit memories (i.e., they did not remember the location of a tunnel in a testing area). When these animals were treated with doxycycline, their ability to form explicit memories returned. Similarly, animals that produced high levels of CaMKII in the amygdala and striatum could not form implicit memories (i.e., they exhibited no fear when they were returned to a testing area where they had previously received a mild electric shock). Again, doxycycline treatment, which abolished CaMKII production, reversed this deficit.

Several conclusions can be drawn from these observations. First, the tTA-dependent regulatory system allows scientists to turn gene X on and off at will. Second, the use of tissue-specific promoters to control tTA production enables researchers to direct the activity of gene X to certain cell types. Because of variations in the site of integration into the mouse DNA, however, individual transgenic mice can differ in the specific cells where gene X is most active. Third, this technology can be used to analyze the role of specific genes in highly complex cognitive processes, such as memory formation, and therefore may also be applicable to studies investigating alcohol’s complex effects on the brain.

## Future Prospects

The technical approaches described in this article have provided scientists with innovative tools for relatively accurately analyzing the functions of individual genes, either by inactivating those genes or by transferring them into other organisms, such as mice. For many applications, however, these methods still are rather crude, because they lead to the activation or inactivation of genes in the wrong cells or tissues or at the wrong times during the organisms’ development. Accordingly, researchers are continually working to further refine these techniques. For example, the Cre-loxP–based gene inactivation system requires the generation of two strains of transgenic mice (i.e., one carrying gene X flanked by the loxP sites, and the other carrying the Cre gene). These strains then need to be mated to generate the transgenic animals in which gene X function can be studied. To simplify this approach, some researchers have used a virus as a vector to introduce the Cre gene into transgenic animals carrying the loxP-flanked gene X, thereby eliminating the need for generating a separate Cre-transgenic mouse strain (Rohlmann et al. 1996; Agah et al. 1997). Similar vectors also can be used to generate mice transgenic for gene X.

The use of a virus as a vector has several advantages. First, the virus can infect adult animals. Thus, when studying genes whose inactivation would interfere with embryonic development, researchers can modify these genes after the crucial developmental period. Second, some viruses are rather specific with respect to the tissues that they infect, thereby allowing researchers to target the Cre protein to those tissues and thus study gene inactivation in them. This tissue-specificity might be enhanced further by placing the Cre gene under the control of a promoter that is active only in the tissue of interest. Third, viruses can effectively deliver the Cre gene, because many viruses infect almost 100 percent of their target cells. Researchers now are attempting to design vectors based on the herpes simplex virus that can specifically infect neurons, including those in the brain, which are particularly difficult to manipulate (Fink and Glorioso 1997). Such vectors might become especially valuable to alcohol researchers, allowing scientists to study alcohol’s effects on the brain in more detail.

Another technique for introducing foreign genes into transgenic animals that is receiving increasing attention involves the use of tiny spheres (i.e., liposomes), whose walls consist of molecules similar to those found in cell membranes (Lee and Huang 1997). The DNA under investigation is introduced into the liposomes’ interior. Once the liposomes have been taken up into a cell, they release the DNA into that cell. Although liposomes are generally less efficient than viral vectors, they have several advantages that make them attractive when generating transgenic animals. First, liposomes are easy to produce in large quantities. Second, it may be possible to target liposomes to specific tissues or cells by attaching other molecules to the liposomes’ surface that are recognized only by specific cells (e.g., certain molecules are recognized only by liver cells). As a result, only the target cells would take up the modified liposomes and would receive the DNA contained within the liposomes. Third, and most important, liposomes, unlike viral vectors, are not toxic to the animals or the target cells and do not induce an immune response. Consequently, liposomes can be used several times to transfer DNA into the same organism.

Other modifications to the existing gene transfer systems strive to improve the investigator’s control over the time and location of the activation or inactivation of gene X. To this end, some researchers have used a “mix and match” approach that combines several of the techniques described in this article. For example, St-Onge and colleagues (1996) have combined the Cre-loxP system used to generate knockout mice with the tetracycline-responsive regulatory system used in transgenic mice. These investigators generated triple-transgenic mice, carrying the tTA gene, the Cre gene under the control of a promoter that is active only in the absence of tetracycline, and gene X containing loxP sites. In this system, gene X was active only if the loxP sites were removed by the Cre protein. Cre protein, in turn, only was produced in the presence of tTA and the absence of tetracycline. Thus, when the animals were exposed to tetracycline, tTA could not activate the promoter controlling the Cre gene, the loxP sites remained in gene X, and gene X was inactive. In the absence of tetracycline, in contrast, tTA activated the promoter of the Cre gene, Cre protein was generated, the loxP sites were excised, and gene X became active. These experiments demonstrated that the tTA (or rtTA) system can be combined with the Cre-loxP system to achieve precise regulation of gene inactivation in knockout mice.

As the examples described in this article indicate, some of these genetic engineering techniques have already been applied in the alcohol field. As researchers identify more candidate genes that may contribute to alcohol’s actions on the body and further refine the methods used to inactivate genes or deliver foreign genes to laboratory animals, these new genetic technologies will be applied to alcohol research with increasing regularity.

## Footnotes

1 For a definition of this and other technical terms used in this article, see glossary, p. 308.

2 Conversely, an immediate hypersensitivity response occurs within minutes after antigen exposure.

## Glossary

Antigen: Any molecule that can induce an immune response.
Chimera: An organism produced by inserting genetic material from one animal into the genetic material of an embryo of another animal. For example, during the generation of *knockout mice*, chimeras are created in which some cells are derived from injected *embryonic stem cells*, whereas other cells are derived from the cells of the embryo into which the embryonic stem cells were injected.
Delayed hypersensitivity response: An immune response that occurs hours to days (rather than minutes) after an organism has been exposed to a foreign *antigen*.
Embryonic stem cell: A type of cell that can develop into all the different cells that make up an intact organism (e.g., blood, nerve, liver, or muscle cells).
Gamma-aminobutyric acid (GABA): A *neurotransmitter* that inhibits the activity of other nerve cells.
Homologous recombination: A process during which a gene introduced into a cell trades places with the corresponding gene in the cell’s DNA.
In vitro studies: Studies performed using cultured cells or tissues.
In vivo studies: Studies performed in intact organisms (i.e., animals or humans).
Knockout mice: Mice in which individual genes have been inactivated.
Liposomes: Small spheres whose walls consist of molecules similar to those found in cell membranes; can be used to introduce foreign DNA into cells.
Marker gene: A gene that is introduced into a cell using a *vector* and which facilitates the identification of cells that have taken up the vector. Marker genes frequently contain the information for producing proteins that confer resistance to certain antibiotics.
Mutation: A change in an organism’s genetic information.
Neurotransmitter: A chemical released by nerve cells that mediates communication among nerve cells or between nerve cells and muscle or gland cells.
Phenotype: The observable characteristics (e.g., physical appearance or behavior) of an organism.
Phosphorylation: A chemical reaction involving the addition of a phosphate group to a molecule (e.g., a protein).
Promoter: A molecular switch that controls the activity of a gene; some promoters are switched “on” all the time, whereas other promoters are switched on only in certain cell types or during specific developmental periods.
Protein kinase: An enzyme that transfers a phosphate group to a protein molecule (i.e., *phosphorylation*).
Receptor: A protein molecule on the surface of a cell that interacts with a specific chemical messenger, such as a *neurotransmitter*.
Serotonin: A *neurotransmitter* that subtly modifies the activity of other nerve cells.
T cell: A type of immune cell that plays an important role during the *delayed hypersensitivity response*.
Transgenic mice: Mice that carry an active foreign gene in addition to their own genes.
Vector: A short piece of DNA that can be easily manipulated and which transfers genes from one organism to another; the DNA’s of viruses are sometimes used as vectors.
Wild-type mice: Normal mice whose genetic makeup has not been altered and who serve as controls in genetic experiments such as those involving *knockout* or *transgenic mice*.
